# Hair-Thread Tourniquet Syndrome: A Comprehensive Review

**DOI:** 10.7759/cureus.60832

**Published:** 2024-05-22

**Authors:** Amr Y Arkoubi, Sajad Ahmad Salati

**Affiliations:** 1 Plastic Surgery, Imam Mohammad Ibn Saud Islamic University, Riyadh, SAU; 2 General Surgery, Unaizah College of Medicine and Medical Sciences, Qassim University, Unaizah, SAU

**Keywords:** acute vascular compromise, child abuse, strangulation, depilatory agent, surgical release, thread, hair strand, hair-thread tourniquet syndrome, hair tourniquet syndrome

## Abstract

Hair-thread tourniquet syndrome (HTTS) is an uncommon but preventable disorder in which a body appendage becomes constricted after becoming firmly wrapped by a hair or substance that resembles hair. The genitalia, fingers, and toes are typically affected. Prompt diagnosis and treatment by complete removal of the constricting agent are crucial for the preservation of the affected appendage. This narrative review article revisits HTTS in the context of the recent literature with the aim of raising healthcare professionals' awareness of this surgical emergency so that the condition can be prevented, correctly diagnosed, and treated early.

## Introduction and background

Introduction & historical background

**Figure 1:**
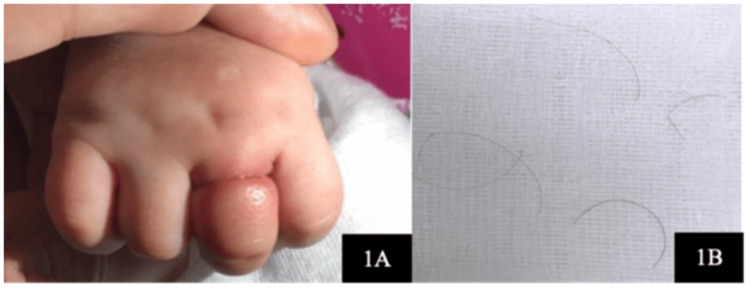
Strangulation over the proximal phalanx of the left ring finger causing edema and congestion (A). Fine hair strands were removed using loupes in the emergency department (B). Image source: Kesu Belani L, Leong JF, Narin Singh PSG, Abdullah S. Hair thread tourniquet syndrome in an infant: emergency exploration saves limbs. Cureus 11(12):e6377. DOI: 10.7759/cureus.6377. Open Access, Creative Commons Attribution-Non-Commercial-No Derivatives License 4.0 (CCBY-NC-ND).

Hair-thread tourniquet syndrome (HTTS) is an uncommon surgical emergency that occurs when a hair, thread, or fiber circumferentially strangulates one or more appendages [1], as depicted in Figure 1.

The condition was first described by Guillimeau in 1612 when he reported strangulation of the glans penis due to hair strands [2]. In 1832, a case was published in the Lancet wherein a "vengeful nurse" having got terminated from her job had intentionally wrapped a hair around the penis of a one-month-old infant [3]. It is conceivable to speculate that the published case was most likely HTTS. In 1888, Morgenstern documented a case of urinary retention and penile edema due to constriction by hair [4]. Quinn described the disorder by using the term “toe-tourniquet syndrome” in 1971 [5].

The term "hair-thread tourniquet syndrome" was coined in 1988 by Barton et al. [6] when they reported a series wherein six cases, ranging in age from 12 days to five months, were treated successfully by immediate removal of the constricting fibers. Various terms have been used to describe this condition, including syndrome of hair tourniquet [2], toe tourniquet [4], hair coil strangulation [5,6], and hair-thread tourniquet syndrome or acquired constriction ring syndrome [7].

Though many case reports have been published in the literature, the syndrome has not received the recognition it deserves and is hardly discussed in textbooks. Consequently, the condition goes unrecognized and misdiagnosed by primary care physicians, leading to serious outcomes [8,9]. The majority of cases are unintentional, but there is always a chance that child abuse may be overlooked as accidental, or else unintentional cases may be wrongly labeled as child abuse, especially when multiple appendages are involved [10]. Given the aforementioned, the purpose of this narrative review article is to raise healthcare professionals' awareness of this surgical emergency so that the condition can be prevented, correctly diagnosed, and treated early, thereby minimizing complications.

## Review

Methods

A manual search was conducted to find observational studies, reviews, and case reports related to HTTS in scientific databases and social networking sites, such as PubMed, Research Gate, Google Scholar, and Web of Science.

The search terms included the following: "hair thread tourniquet syndrome," "hair tourniquet," "hair coil strangulation," "toe tourniquet,” “acquired constriction ring syndrome,” “management,” and "complications.” A timeframe of 2000 to 2024 was chosen to concentrate on the recent literature, and only the English-language publications were assessed. However, several cross references from prior to 2000 were also included as they provided insight into the historical facets of this disorder.

The inclusion criteria for the article type were original articles, case reports, case series, and review articles. Editorials and letters to the editor were excluded. The factors that were focused upon during the analysis of the literature included epidemiology, pathophysiology, clinical features, classification, predisposing factors, treatment, complications, and awareness levels about this condition. The data were independently extracted by the authors and then after a comparison of notes, the sections of the articles were compiled. The writers separately extracted the data, and then they compared their notes to build up the article's subsections.

Epidemiology

The majority of HTTS cases are in newborns and young children, and there is no gender-based disparity in HTTS incidence. The site involved varies with the age of the patient. According to Barton et al. [6], patients' digits are often involved up to 1.5 years, whereas penile involvement occurs between four months and six years of age. Children in the older age range of seven to 13 years have been more affected by HTTS involving the genitalia [11].

Claudet et al. [12], after an analysis of 57 cases, discovered that in 58% of cases, the parents were well known for their neglectful behavior and lived in a state of extreme poverty in dilapidated housing.

Pathophysiology

There is a lack of consensus on the precise mechanism by which the hair strands get wrapped around an appendage so snugly, though the repetitive movement of the appendage in a confined area (e.g., hand in mitten) is one suggested possibility. Hair primarily affects the toes, whereas loose threads (e.g., from mittens) encircle the fingers in particular. The third toe is the most frequently involved digit, but the exact cause is unknown [2].

When hair is wet, it tends to be pliable and stretched out, but when it dries, it contracts, which is why it acts as a tourniquet when coiled around an appendage. Additionally, the whorled arrangement promotes hydrogen-bond formation, which imparts a greater tensile strength of more than 29,000 pounds per square inch to the structure [13,14].

**Figure 2:**
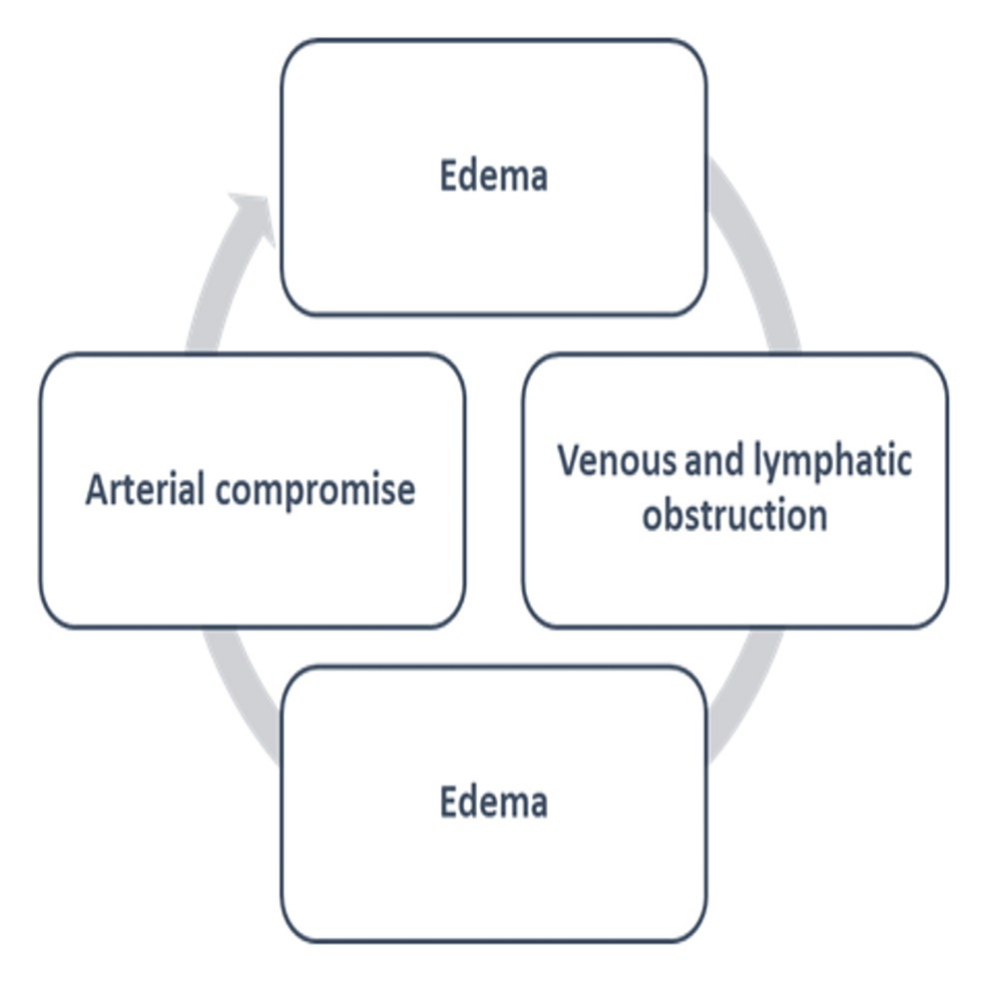
Pathophysiology of hair-thread tourniquet syndrome. Image credits: Amr Y. Arkoubi and Sajad Salati (authors).

The constricting band impedes lymphatic and venous outflow, resulting in edema formation with raised interstitial pressures. The band becomes increasingly tighter and more concealed within the edematous, entangled portion, making diagnosis and identification more challenging. The arterial supply to the region distal to the constriction steadily decreases, leading to ischemia [15]. This vicious cycle is set in motion and perpetuated by the increasing edema, as shown in Figure 2. A secondary infection may set in and further complicate the situation. Similarly, granulation tissue formation and re-epithelialization bury the constricting bands, making diagnosis difficult [15]. This process may take several days or even weeks to complete [16].

Clinical presentation

**Figure 3:**
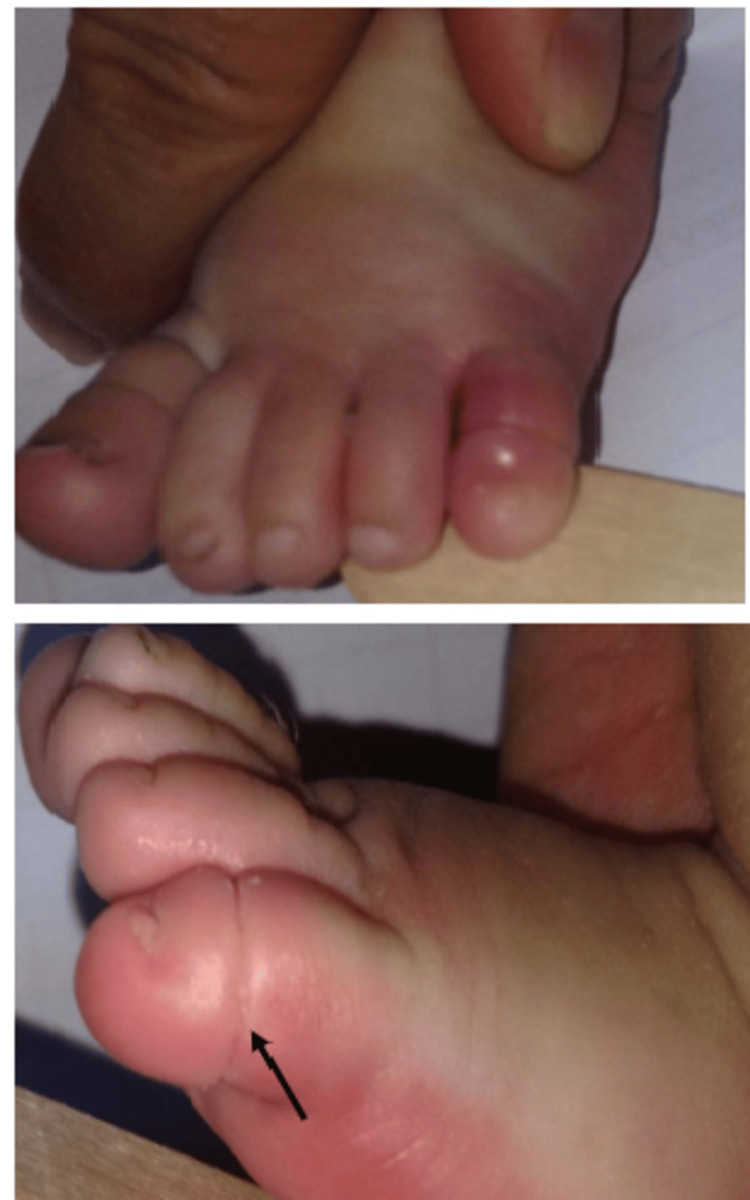
Gross swelling of the 5th toe with purple discoloration and circumferential tissue cleft. Image source: Ugur LOK, Gulacti U, Borta T, Celik M, Aktas N, Buyukaslan H. Hair-thread tourniquet syndrome: a presentation of an infant. Arc Case Rep Clin Med. 2016; 2(3):124. DOI: 10.19104/crcm.2016.124. Open Access, Creative Commons Attribution-Non Commercial-No Derivatives License (CCBY-NC-ND).

**Figure 4:**
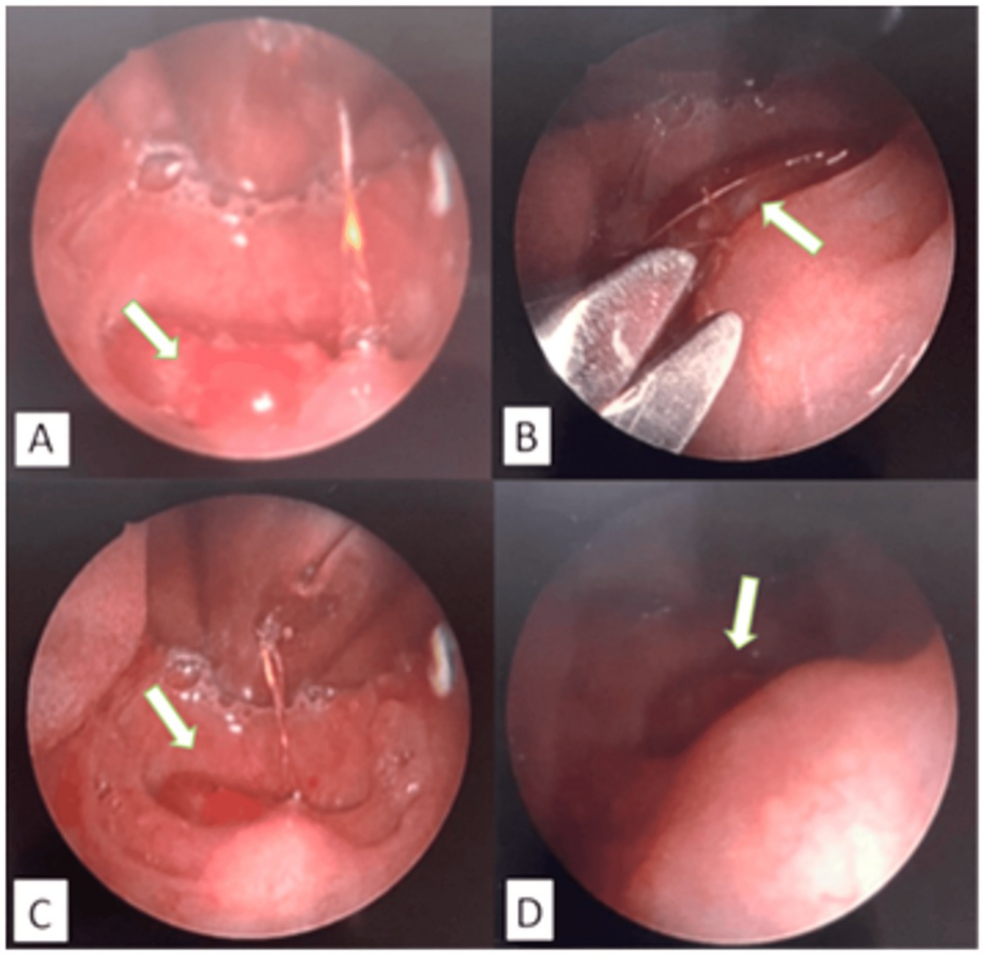
Images of hair tourniquet on the uvula and the removal of the foreign body. (A) Endoscopic images of hair tourniquet on the uvula. (B) Surgical removal of hair fiber with iris scissors. (C) Uvula immediately after removal; engorgement and edema of the uvula can still be seen. (D) Uvula five minutes after the removal; edema improved significantly. Image source: Schneider TA, Ahluwalia J, Mnatsakanian A, Haupert M. Hair tourniquet syndrome involving the uvula secondary to an airway foreign body. Cureus 16(2):e53656. DOI: 10.7759/cureus.53656. Open Access, Creative Commons Attribution-Non Commercial-No Derivatives License (CCBY-NC-ND).

A child may be agitated and inconsolably crying during presentation [17]. A thorough physical examination reveals a painful, swollen appendage that is clearly and circumferentially delineated from the proximal normal tissue [18], leading to the diagnosis, as depicted in Figures 1, 3, 4. The appendage may either be frankly autoamputated or else gangrenous, dry, and shriveled up if the presentation is significantly delayed [19].

**Figure 5:**
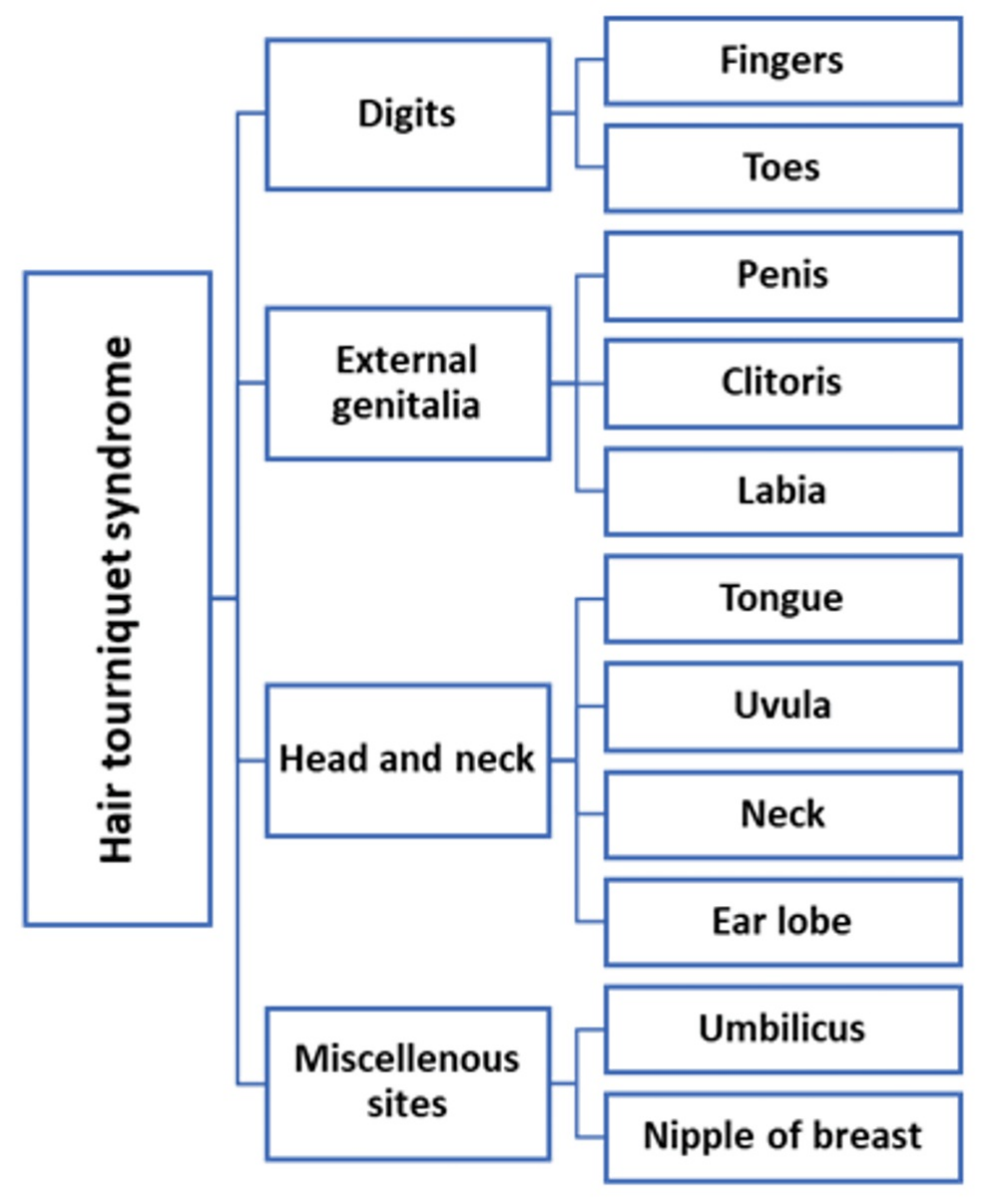
Body parts affected by hair tourniquet syndrome. Image credits: Amr Y. Arkoubi and Sajad Salati (authors).

The structures that have been reported to be affected by HTTS in literature reviews are as depicted in Figure 5, with digits and external genitalia making up the majority in most of the series [20]. In an extensive literature review and meta-analysis conducted by Mat Saad et al. [21], comprising 210 cases of HTTS, the affected appendages in decreasing order of frequency were penis (44.2%), toes (40.2%), fingers (8.6%), and other miscellaneous sites (6.8%). Bean et al. [22] retrospectively reviewed 81 patients with HTTS, ranging in age from two weeks to 22 years. They found that 69 were located on the toes, five on the fingers, and seven on the genitalia.

Unusual Presentations

HTTS presenting in unusual ways with atypical sites or patterns of injury are reported in the literature. In penile HTTS, a ventral urethrocutaneous fistula proximal to the construction was reported by Bangroo and Chauhan in a 10-year-old child [23].

Okeke [24] reported a nine-year-old child who had been straining during micturition for three years and had minimally discharging non-healing wounds over the dorsolateral penis. On physical examination, there was palpable periurethral induration and a circumferential scar over the midshaft of his penis. A tight stricture was found in the middle third of his urethra in imaging studies. There was no discoloration or alteration of sensation distal to the scar, and this was speculated to be due to the ligature not being too tight to compromise circulation but tight enough to induce pressure necrosis. Surgical exploration revealed an intact black hair thread tourniquet embedded in dense fibrous tissue and associated with a 2 cm-long dense urethral fibrosis. The tourniquet was released, the fibrotic urethra excised, and a single-stage substitution urethroplasty based upon a distal penile pedicled skin flap was performed. At 28 months of follow-up, the patient has been voiding well.

Kumaravel [25] reported a rare and delayed presentation of HTTS in a two-year-old child as a non-healing, discharging ulcer. The fibrotic bands were identified with the help of loupes and removed completely. Mat Saad et al. [21] presented a unique case of a three-month-old baby girl in whom a hair cheese-wired through the skin and soft tissue of the toe to reach the underlying phalangeal bone and caused its erosion. Rampersad et al. [26] reported a two-year-old uncircumcised male who presented with one day of erythema to the base of the penis, followed by a bleeding wound of five hours duration. On examination, the shaft skin at the base of the penis was circumferentially separated, and examination of the diaper revealed a coiled strand of maternal hair. Debridement followed by primary repair was conducted, with an uneventful recovery.

Hair-Thread Tourniquet Syndrome in the Elderly

Cases of HTTS have been reported in the elderly, particularly those with cognitive impairment. Srinivasaiah et al. [27] reported an 80-year-old male with dementia who had a dusky and swollen inferior third of a leg distal to a circumferential zone with patchy areas of granulation. A careful examination revealed rubber bands embedded in the granulation tissue. Miller et al. [28] reported HTTS involving the fourth toe in an 84-year-old nursing home resident suffering from Alzheimer’s disease.

Predisposing factors

Various predisposing factors have been identified for HTTS, which include the following.

Telogen Effluvium

This is the excessive shedding of hair that most women experience during the postpartum period and is caused by hormonal changes in the body. This stage may last for two to six months following delivery, and that is the phase when there is the maximum probability of suffering from HTTS. Understanding this connection is important because it may help prevent cases of toe tourniquet syndrome and the morbidity that goes along with it if new mothers who are experiencing rapid hair loss receive proactive counseling [29].

Gratification Disorder or Infantile Masturbation

This may be a factor leading to genital HTTS, and this factor has high significance due to the fact that this behavior is displayed by 90-94% of males and 50-60% of females [2].

Co-sleeping and Bed Sharing

This is a common cultural practice in Asian families, where parents sleep in close proximity to their infants. Murata and Sakakibara [30] have reported HTTS of the neck in a two-year-old boy who would co-sleep with a sister who had long, curly hair and presented with irritability, crying, and swelling over the neck. Physical examination revealed a long hair-thread tourniquet encircling his neck and compressing the underlying tissues. There were periocular petechiae, but there was no respiratory distress or any neurological deficits.

Circumcision

This has been linked to HTTS, as the constricting bands tend to get wrapped around the exposed coronal sulcus of a circumcised child [8].

Cultural Practices

Many cultural practices have been mentioned as risk factors for HTTS in the literature. It is well known that gypsies weave hair around people's fingers and toes, both young and old, in an attempt to ward off evil spirits [31]. In certain African cultures, wrapping hair around the nipple is one method of weaning infants off breastfeeding [31]. Similarly, the wrapping of a band, ribbon, or hair around the penis is deliberately done to stop nocturnal enuresis. In certain tribes, a strand of hair is wrapped around the base of the penis of babies with the belief that adult sexual function will improve [31].

Level of Hygiene & Poverty

Lack of proper hygiene and poverty have been mentioned as risk factors. Claudet et al. [12] analyzed 57 children with HTTS with a mean age of 5.5 ± 4 months and discovered improper hygiene in 67%.

Differential diagnosis

HTTS can mimic a variety of disorders that present acutely with pain, swelling, and/or erythema, including trauma, cellulitis, insect stings, acute paronychia, and allergic or non-allergic dermatitis. In regions with cold and harsh winters, pernio (chilblains) is an important differential diagnosis. Bhat et al. [32] from Kashmir Valley have reported a series of 10 infants who were treated for impending gangrene of fingers in infants due to HTTS resulting from mother’s hair, and before presentation, all of them had been misdiagnosed as pernio.

Similarly, HTTS needs to be differentiated from disorders causing skin thickening, like palmoplantar keratoderma, and from disorders that tend to form constriction bands, like congenital constriction bands and ainhum (dactylolysis spontanea) [2,10].

Relationship with child abuse/child neglect

The possibility of child abuse or neglect should be considered while handling a case of HTTS [12]. Klusmann and Lenard [33] presented five patients with HTTS, four of whom involved the toes, and in one, the clitoris was strangulated. The meticulous wrapping of constricting material, combined with the lack of any plausible explanation, increased the likelihood that the course was not accidental. Involvement of multiple appendages, multiple stands of hair with multiple tight knots, constrictive agents inconsistent with a safe environment for the child, and ecchymosis or signs of fresh or healed injuries should raise suspicions about child abuse [33].

Relationship between the affected part, patients’ age, and etiology

Kursad et al. [34], on the basis of a literature review, tried to figure out the relationship between the location of the lesion, the age of the patient, and the possible causative factor in HTTS. They found that penile strangulation in infancy is generally accidental and occurs as a result of the mother's hair (telogen effluvium). In children, especially between four and 11 years, the age that corresponds to Sigmund Freud’s phallic phase of psychosexual development, often the thread fabrics instead of hair, are the causative elements in HTTS. In adults, HTTS may be self-inflicted, resulting from the use of materials like condom rings to enhance sexual experiences. They further suggested that the lesions in the proximal half of the penis are most likely self-inflicted rather than accidental, and in many cases, to address some other disorder like nocturnal enuresis. They also stressed that identification of the causative element would help in the prevention of further episodes.

Grading and classification

**Figure 6:**
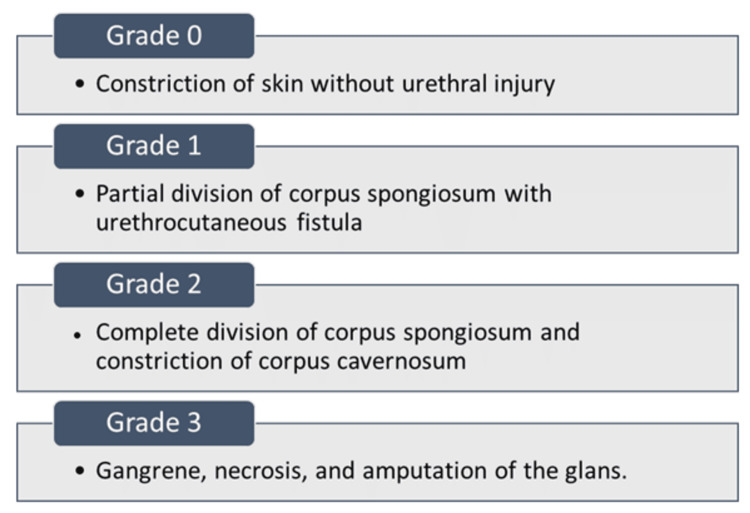
Grading of penile hair-thread tourniquet syndrome. Image credits: Amr Y. Arkoubi and Sajad Salati (authors).

Attempts have been made to grade the degree of injury due to HTTS, and the grading system for penile HTTS (Figure 6) by Bashir and El-Barbary [35] has gained wide acceptance and application.

Role of imaging in diagnosis

According to Sebaratnam and Hernandez-Martin [36], ultrasonography may be able to provide a timely and accurate diagnosis and may be a helpful supplement to clinical evaluation in HTTS, particularly when the constricting band is concealed. In their presented case, bedside high-resolution ultrasonography revealed a thicker epidermis covering a hyperechoic focus inside the dermis in a two-year-old child who had presented with a two-month history of erythematous, indurated plaque on the third toe of her right foot with well-defined borders. Her sonographic and clinical appearance were consistent with HTTS, and the constricting bands were surgically removed without any adverse neurovascular sequelae.

Treatment

The treatment of HTTS primarily consists of decompression and complete release of the constricting bands as early as possible after diagnosis. This can be achieved by non-surgical or surgical approaches, and the adoption of choice depends upon the depth, nature, and visibility of the constricting bands [37].

Non-surgical

For superficially positioned and visible constricting bands comprised of hair but not thread, the employment of a depilatory agent with a thioglycolate base is a very effective and painless technique [38]. A small amount of depilatory agent is applied as cream or foam, followed by a wait of three to 10 minutes to allow the breakdown and dissolution of the hair. The area is then carefully cleaned. The relative contraindications include allergies to the constituents of depilatory creams, mucosal surfaces, and non-intact skin [38]. Bean et al. [22] successfully treated 65 out of 69 (94%) cases of HTTS with this technique. Alruwaili et al. [38] also reported painless treatment in two cases. Kudzinskas et al. [39], after reviewing 19 articles from peer-reviewed literature, found that 64% of patients had successful resolution of their HTTS after one or two cycles of depilatory agent treatment.

Surgical

Surgical incision with the exploration of the area under proper anesthesia is the technique of choice for deeply embedded constrictors and in the presence of edema [38]. For embedded constricting bands and in the presence of edema, the preferred treatment is a surgical exploration under adequate anesthesia. Less intrusive methods can be tried if there is little edema and the thread is visible. For example, using a blunt probe to cut the thread directly or unwinding the thread. Nevertheless, to remove deeply embedded bands, a longitudinal surgical incision should be made deep enough to achieve complete release [38]. Reconstruction, if any, depends upon the nature of the injury.

Tamborini et al. [40] recently presented the successful application of the "Hirase technique" after surgical release for salvaging a compromised toe in HTTS. The forefoot was wrapped in aluminum foil, and an ice pack was placed around it as soon as the thread was safely cut and removed. Three days later, the toe was fully intact and showed no signs of necrosis. After six months, the skin of the damaged digit had fully recovered, the perfusion was normal, and there was no onychopathy.

Awareness and attitude toward HTTS

Awareness about HTTS is lacking, and activities to enhance awareness levels would help healthcare providers identify and treat the condition promptly. Some simple prevention strategies should be popularized, and they can help parents and other caregivers mitigate risk [41]. Biehler et al. [42] evaluated the expertise of professionals in the disciplines of child welfare, nursing, and medicine through a survey. Following the presentation of a case history and several typical HTTS photographs, the participants were asked to interpret the injuries. Of all respondents, almost half indicated they would report the injuries as possible child abuse. A significantly higher percentage of child welfare workers (83%), compared to public health nurses (45%), stated that the injuries were suggestive of abuse. However, there are no published studies in the peer-reviewed literature that could have pointed out the impact of efforts aimed at raising awareness about HTTS. Such initiatives must be carried out, and the outcomes must be reported.

Prevention

During the antenatal phase, the expectant parents should be made aware of HTTS and the prevention strategies. And, when HTTS is treated, parents should be counseled to ensure close follow-up for re-examination so that deformities may be detected early and possible reconstruction planned [42].

Limitations/potential biases

This article was primarily drafted after analysis of literature that was either made available by the Saudi Digital Library through institutional subscriptions or that was obtained from the Open Access category. Data omissions may have occurred if certain information was excluded from these two sources. That being said, it is worth noting that Saudi Arabian scholars have access to a substantial number of publications via the Saudi Digital Library, therefore it is unlikely that the number of unavailable papers would be substantial.

## Conclusions

HTTS is an uncommon, potentially serious, but preventable disorder that primarily affects neonates. Early diagnosis and treatment are essential for a favorable outcome and to prevent further injury to the patient. To that purpose, parents and healthcare professionals should be aware of the symptoms and indicators of this illness. Options for management could involve surgery or non-surgical procedures, depending on the type of lesion.
